# Singapore COVID-19 data cross-validation by the Gaidai reliability method

**DOI:** 10.1038/s44298-023-00006-0

**Published:** 2023-12-13

**Authors:** Oleg Gaidai, Vladimir Yakimov, Jiayao Sun, Eric-Jan van Loon

**Affiliations:** 1 Shanghai Ocean University, Shanghai, China; 2 Jiangsu University of Science and Technology, Zhenjiang, China; 3 Central Marine Research and Design Institute, Saint Petersburg, Russia; 4 Ghent University, Ghent, Belgium

**Keywords:** Computational biology and bioinformatics, Medical research

## Abstract

Novel coronavirus infection (COVID-19) has exserted certain burden on global public health, spreading around the world with reportedly low mortality and morbidity. This study advocates novel bio and health system reliability approach, especially suitable for multi-regional environmental and health systems. Advocated spatiotemporal method has been cross-validated, versus well established bivariate Weibull method, based on available raw clinical dataset. The purpose of this study was to assess risks of excessive coronavirus death rates, that may occur within any given time horizon, and in any region or district of interest. This study aims at benchmarking of the novel Gaidai bio-reliability method, allowing accurate assessment of national public health system risks, for the years to come. Novel bio-system reliability approach is particularly suitable for multi-regional environmental and health systems, monitored for a sufficiently representative period of time. In case when underlying bio-system is stationary, or the underlying trend is known, long-term future death rate risk assessment can be done, and confidence intervals can be generated. Advocated methodology may to be useful for a wide variety of public health applications, thus, it is not limited to the example, considered here.

## Introduction

Statistical characteristics of COVID-19 (SARS-CoV-2) and other comparable recent influenza outbreaks have been receiving substantial research interest in recent years^1–3^. Environmental effects on biological systems typically follow cyclical patterns. For environmental effects see ref. ^4^; for meteorological parameters see ref. ^5^; for heat stress and thermal perception see ref. ^6^. In general, determining actual biological system’s reliability factors to assess future epidemic outbreak risks, is fairly challenging, given a variety of epidemic and environmental factors. In principle, direct MC (Monte Carlo) simulations or a sufficient number of raw clinical observations might be sufficient to evaluate reliability of a complex biological system. However, COVID-19’s clinical observational data are limited to years 2020–2022. In order to address the challenge of having too limited underlying clinical dataset, the authors have developed Gaidai reliability approach, suitable for biological and health systems, when risks of near future epidemic outbreaks are of interest. COVID-19 outbreaks in Singapore were the primary focus of this study, which focused on cross-correlations between various health data from the same climatic zone. Singapore has been chosen due its extensive national health surveillance, and its publicly accessible raw clinical data^7^. Engineering and medical research both make extensive use of statistical lifetime data modeling by EVT (extreme value theory)^8^. In ref. ^9^ EVT has been utilized by authors to forecast H1N1 (swine flu) epidemiological risks. For spatial lag and error models, along with regression techniques, see ref. ^10^. In the current study an epidemic outbreak is defined as unexpected random event, that might occur at any time and in any administrative region of a particular national health care system. Spatiotemporal aspect of epidemiological risk has been therefore taken into account. Non-dimensional parameter *λ* has been introduced to unify various national regions with different epidemiological backgrounds into one multi-dimensional bio-dynamic system.

Singapore’s COVID-19 raw clinical data has been retrieved from a public source^7^. National public health system under investigation has been modeled as MDOF (multi-degree-of-freedom) dynamic biosystem, with strongly correlated administrative components (spatial dimensions). The goal of this study was assessment of future epidemiological outbreaks risks, hence authors only considered daily reported patient numbers, and not symptoms. The map of Singapore represents specific clinical recorded instances.

Based on quasi-stationarity assumption, this study assumed that, despite seasonal fluctuations, the underlying epidemiological process would be statistically representative throughout two consecutive observational years, 2020–2022. In case of underlying trend is of interest, it should be identified first, and epidemiological thresholds should be made variable with time. In the latter case, Gaidai-Yakimov method can be applied even to non-stationary bio-systems.

## Method

MDOF dynamic system is represented here by a collection of its critical/key components/dimensions, combined into biosystem’s representative vector$\left(X\left(t\right),Y\left(t\right),Z\left(t\right),\ldots \right)$, consisting of biosystem’s key components $X\left(t\right),Y\left(t\right),Z\left(t\right),\ldots$ that has been measured/observed over sufficiently long (representative) clinical period $(0,T)$. Biosystem component’s global maxima being denoted as ${X}_{T}^{\max }=\mathop{\max }\limits_{0\le t\le T}X\left(t\right)$, ${Y}_{T}^{\max }=\mathop{\max }\limits_{0\le t\le T}Y\left(t\right)$, ${Z}_{T}^{\max }=\mathop{\max }\limits_{0\le t\le T}Z\left(t\right),\ldots$. By sufficiently long clinical/observational duration $T$ authors primarily mean long enough observational duration $T$ with respect to the dynamic bio-system relaxation and auto-correlation time scales. Let ${X}_{1},\ldots ,{X}_{{N}_{X}}$ be temporally consequent biosystem component $X=X(t)$ local maxima occurring at discrete temporally non-decreasing time-instants ${t}_{1}^{X} < \ldots < {t}_{{N}_{X}}^{X}$ within clinical observational period$(0,T)$. Identical definitions can be given for other MDOF bio-system’s key components $Y\left(t\right),Z\left(t\right),\ldots$ namely ${Y}_{1},\ldots ,{Y}_{{N}_{Y}};$ ${Z}_{1},\ldots ,{Z}_{{N}_{Z}}$ and so on. For simplicity, all biosystem components, and hence their local maxima have been assumed to be positive. Hence:

1 $$\begin{array}{l}P=\mathop{\iiint }\nolimits_{\left(0,0,0,,\ldots \right)}^{\left({\eta }_{X},{\eta }_{Y},{\eta }_{Z},\ldots \right)}{p}_{{X}_{T}^{\max },{Y}_{T}^{\max },{Z}_{T}^{\max },\ldots }\left({x}_{T}^{\max },{y}_{T}^{\max },{z}_{T}^{\max },\ldots \right)\\ \qquad {d{x}_{T}^{\max }d{y}_{T}^{\max }d{z}_{T}^{\max }\ldots}\end{array}$$

representing bio-system’s survival probability $P$, given in terms of joint PDF (probability density function) $p$. Due to biosystem’s high dimensionality, it is not practical to assess ${p}_{{X}_{T}^{\max },{Y}_{T}^{\max },{Z}_{T}^{\max },\ldots }$ directly. When either of key component $X\left(t\right)$ exceeds ${\eta }_{X}$, or $Y\left(t\right)$ exceeds ${\eta }_{Y}$, or $Z\left(t\right)$ exceeds ${\eta }_{Z}$, etc., biosystem is viewed as having instantly failed or entered in a state of hazard. Fixed hazard/failure levels ${\eta }_{X}$, ${\eta }_{Y}$, ${\eta }_{Z}$,… being individually set for each 1D (1-dimensional) biosystem’s component. The latter target biosystem survival probability $P$ is needed to assess biosystem’s expected lifetime. Bio-system’s 1D key components $X,Y,Z,\ldots$ being now re-scaled as well as non-dimensionalized:

2 $$X\to \frac{X}{{\eta }_{X}},Y\to \frac{Y}{{\eta }_{Y}},Z\to \frac{Z}{{\eta }_{Z}},\ldots$$

making all bio-system key components non-dimensional, having identical failure/hazard limits, equal to 1. Synthetic temporally non-decreasing vector being now created by merging/coalescing biosystem component’s local maxima into 1D combined system vector $\vec{R}=\left({R}_{1},{R}_{2},\ldots ,{R}_{N}\right)$ coherent with corresponding combined temporal vector ${t}_{1}\le \ldots \le {t}_{N}$, $N\le {N}_{X}+{N}_{Y}+{N}_{Z}+\ldots$. Each biosystem’s key component local maxima, constituting vector ${R}_{j}$ being actually observed within biosystem temporal record, occurring within either $X\left(t\right)$ or $Y\left(t\right)$, or $Z\left(t\right)$ or other biosystem’s components. Constructed synthetic $\vec{R}$-vector has 0 data loss.

Now temporally non-decreasing synthetic vector $\vec{R}$, along with its corresponding component’s occurrence time instants ${t}_{1}\le \ldots \le {t}_{N}$, have been now fully introduced^11–13^.

## Results

This section utilizes advocated approach to a bivariate random bio-process $Z(t)=(X(t),Y(t))$ to demonstrate its efficiency. Patients with COVID who have been diagnosed and daily records are included in this approach, $X(t),Y(t)$, being monitored synchronously over a certain observational time span $(0,T)$. It being assumed for simplicity that samples $({X}_{1},{Y}_{1}),\ldots ,({X}_{N},{Y}_{N})$ within observational time period $\left(0,T\right)$ were collected at *N* equidistant discrete time instants ${t}_{1},\ldots ,{t}_{N}$^11,12,14,15^$,$ yielding bivariate joint CDF $P\left(\xi ,\eta \right):={\rm{Prob}}\left({\hat{X}}_{N}\le \xi ,{\hat{Y}}_{N}\le \eta \right)$ of the 2D vector $\left({\hat{X}}_{N},{\hat{Y}}_{N}\right)$, with components ${\hat{X}}_{N}=\max \left\{{X}_{j}{\rm{;}}j=1,\ldots ,N\right\}$, and ${\hat{Y}}_{N}=\max \left\{{Y}_{j}{\rm{;}}j=1,\ldots ,N\right\}$. In doing so, it serves as an example of a dynamic two-dimensional (2D) system^12,13,16^. Using one-dimensional extreme response values with return times and probabilities, critical thresholds were found $p$. Scaling has been done to combine both time series $X,Y$ in accordance with Eq. (2), resulting in each of the two bio-system components having failure/hazard unitary limit equal to 1. Then, by maintaining them in temporal non-decreasing order, all biosystem components local maxima from each measured system component time-series have been combined into one single time-series $\vec{R}=\left(\max \left\{{X}_{1},{Y}_{1}\right\},\ldots ,\max \left\{{X}_{N},{Y}_{N}\right\}\right)$.

### Synthetic environmental example

The authors selected synthetic example, where exact analytical solution is known in advance. The latter made it possible to cross-validate advocated reliability method versus well established bivariate Weibull method. Note that Gaidai method can tackle high-dimensional systems, while bivariate Weibull method is suitable only for 2D (2-dimensional) systems. The latter is a distinctive advantage of Gaidai method.

Wind speed 3.65-day maxima process $X\left(t\right)$ has been modeled within time period $\left[0,T\right]$, based on stationary underlying Gaussian stochastic process $U\left(t\right)$, having zero mean value and standard deviation equal to 1. It was assumed for simplicity that $U\left(t\right)$ mean zero up-crossing rate equals ${\nu }_{U}^{+}\left(0\right)={10}^{3}/T$, with return period $T=1$ year^14,15,17–19^. As a result, wind speed maxima process $X\left(t\right)$ will have 365/3.65 = ${10}^{2}$ data points annually, with total data record containing ${10}^{4}$ data points, which being equivalent to 100 years. Underlying wind speed process $U\left(t\right)$ has 3.65 days maxima analytical CDF (cumulative density function) ${F}_{X}^{3d}\left(x\right)=\exp \left\{-q\exp \left(-\frac{{x}^{2}}{2}\right)\right\}$ corresponding to the 3 days wind speed maxima process ${X}^{3d}\left(t\right)$. Gumbel-Haugaard, Frank, and Clayton are three Archimedean copulas that are often used. The Gumbel-Haugaard copula $G\left(u,v\right)$ dependence structure being taken into account initially, modeling cross-correlation between the marginal peak wind speed variables ${X}^{3d}\left(t\right)$ and symmetrically distributed cross-correlated process ${Y}^{3d}\left(t\right)$:

3 $$G\left(u,v\right)=\exp \left\{-{\left[{\left(-\log u\right)}^{m}+{\left(-\log v\right)}^{m}\right]}^{\frac{1}{m}}\right\}$$

with ${X}^{3d}\left(t\right)$ and ${Y}^{3d}\left(t\right)$ having correlation coefficient ${R}_{{{\rm{corr}}}}$ of 0.5, and parameter ${m}=1/\sqrt{1-{R}_{{{\rm{corr}}}}}$ being connected to correlation coefficient ${R}_{{{\rm{corr}}}}$. Since stationary random Gaussian processes underlying both ${X}^{3d}=X\left(t\right)$, and ${Y}^{3d}=Y\left(t\right)$, the Gumbel-Haugaard copula is easily adaptable, hence bivariate Weibull method prediction agrees well with both analytical solution *x* = 6, as well as with Gaidai prediction. Exact bivariate CDF reads as:

4 $${H}^{3d}\left(x,y\right)=\exp \left\{-{\left[q\exp \left(-m\frac{{x}^{2}}{2}\right)+q\exp \left(-m\frac{{y}^{2}}{2}\right)\right]}^{\frac{1}{m}}\right\}$$

**Fig. 1:**
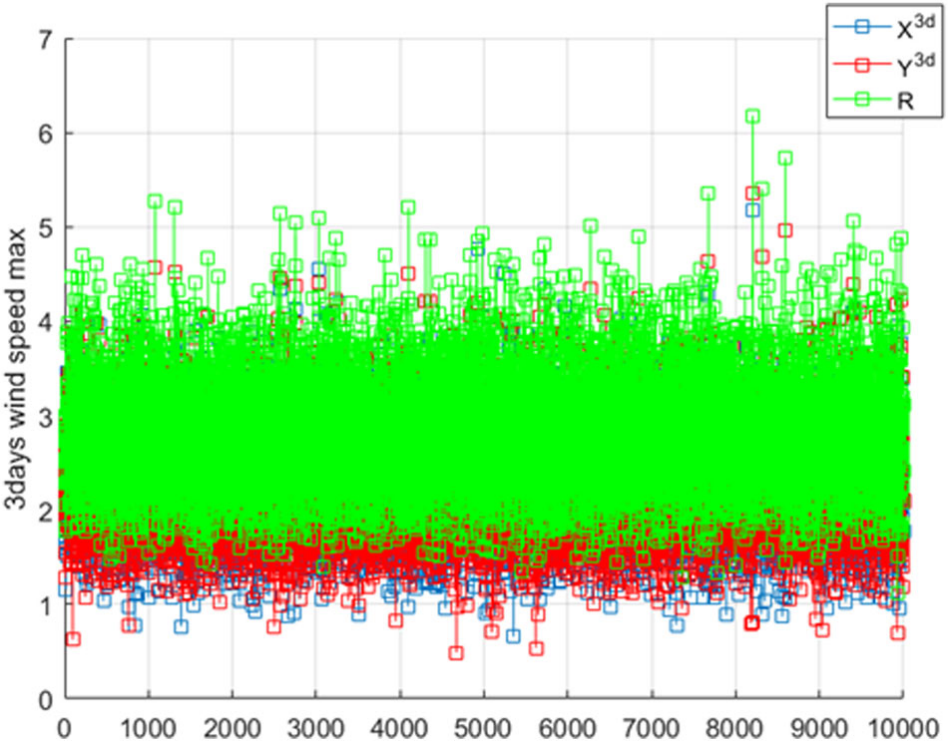
Raw synthetic timeseries along with corresponding $\vec{{\boldsymbol{R}}}$ vector^13^. Blue and red squares - two original system components X and Y; green squares - synthetic merged vector R.

Figure 1 presents simulated (synthetic) time series, coalesced into 1D system $\vec{R}$ vector. Bivariate Weibull contour, with target probability level 2D contour, containing selected bivariate test-point $\left({X}^{3d},{Y}^{3d}\right)=\left(\mathrm{6,5.2}\right)$ agreed well with both analytical and Gaidai method’s prediction $R=6$, as expected, since underlying stochastic process was rather simple. Second, the equivalent Clayton copula was used in place of Gumbel-Haugaard copula $C\left(u,v\right)$, with asymmetric Archimedean copula:

5 $$C\left(u,v\right)=\max \left\{{\left[{u}^{-m}+{v}^{-m}\right]}^{-\frac{1}{m}},0\right\}$$

Clayton copula being more challenging for bivariate Weibull method to fit, since it being not part of the copula library—currently implemented are only Asymmetric logistic and Gumbel logistic copulas^20–26^. Bivariate Weibull method being therefore expected to perform less accurately than Gaidai method in this case.

For specific numerical example mentioned above, it was found that, on average, Gaidai method performed 15–20% more accurately than bivariate Weibull technique. In the case of raw measured non-Gaussian, cross-correlated by non-Archimedean copulas data, an advantage of Gaidai method would be more pronounced. Last but not least, bivariate Weibull clearly required more processing time than Gaidai approach for any given bivariate failure/hazard limit since it performs 2D surface interpolation. Gaidai method has produced 95% CI (confidence interval), while bivariate Weibull method did not have such ability.

### Method validation

**Fig. 2:**
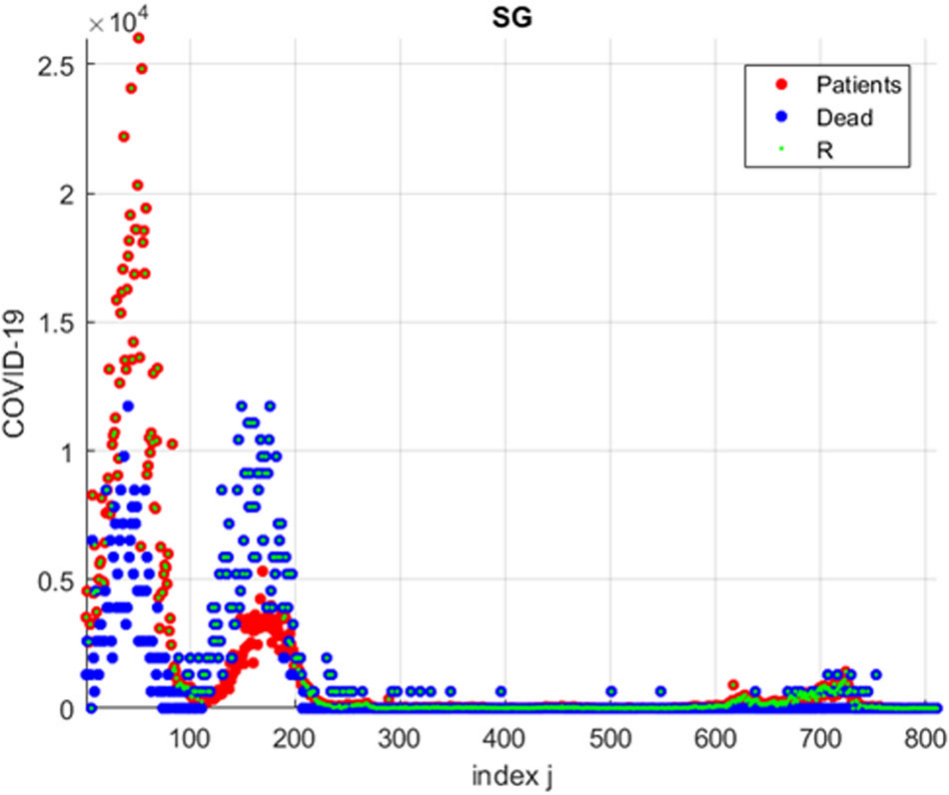
Example of Singapore COVID-19 patient’s data, presented as daily observed raw timeseries. Red and blue circles - two original bio-system components (number of recorded and dead patients); green squares - synthetic merged vector R.

Figure 2 presents an example of Singapore COVID-19 raw clinical death rate data, recorded during the years 2020–2022, presented as observed timeseries.

**Fig. 3:**
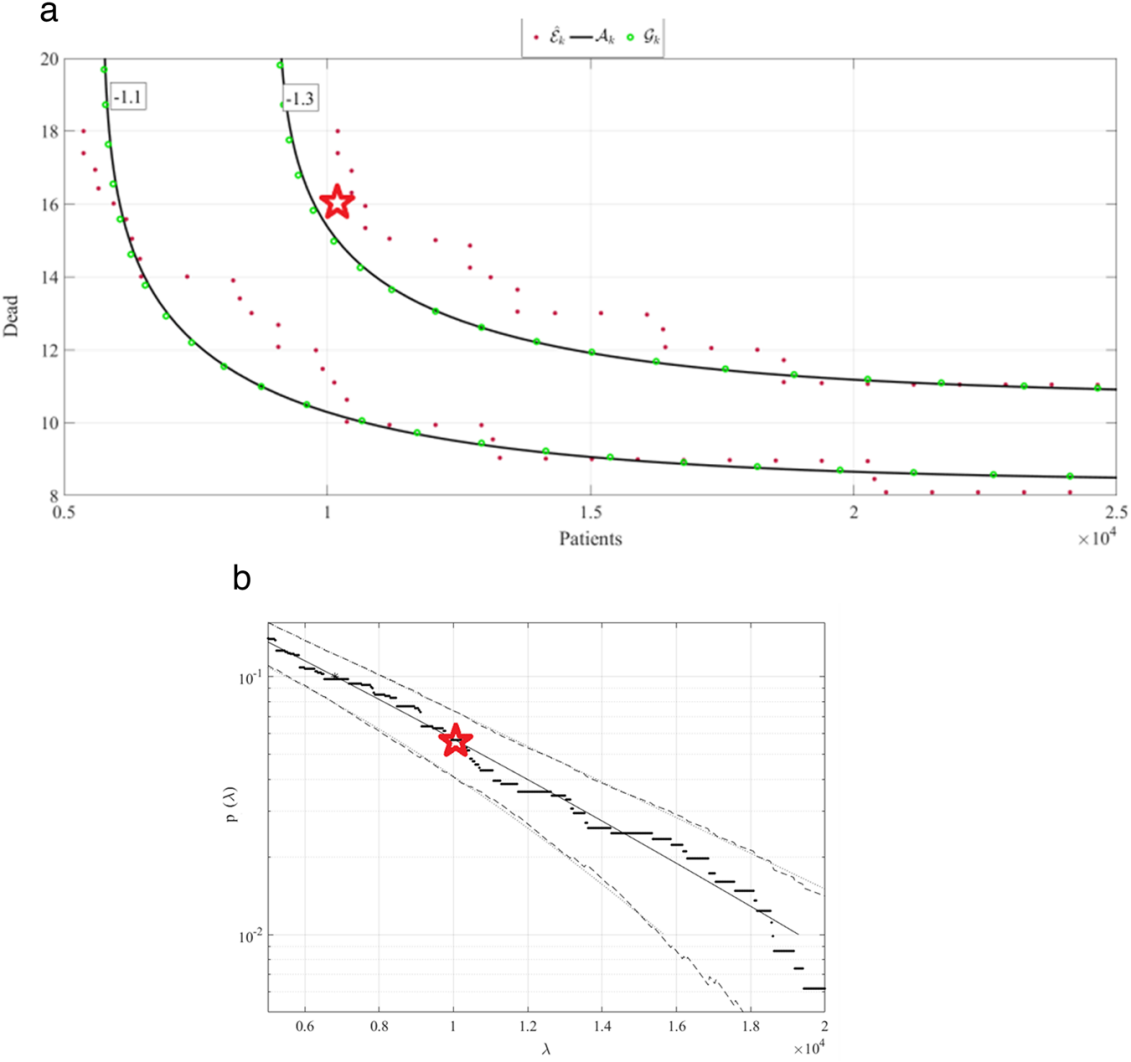
Gaidai method validation versus bivariate Weibull method. **a** Bivariate Weibull bivariate contours for Singapore COVID-19 data. **b** Gaidai prediction star indicates the same bivariate failure level of interest.

Figure 3 presents bivariate Weibull bivariate contours for Singapore COVID-19 death rate data^7^. As seen from Fig. 3, there is an intrinsic inaccuracy, owing to the specific copula choice within bivariate Weibull fit to the raw measured dataset. See for more information on the bivariate Weibull technique^19,20^. Bivariate failure/hazard test-point $\left(X,Y\right)=\left({44,000,\,65}\right)$ has been selected for comparison between two methods (Gaidai and bivariate Weibull), as this bivariate test-point lies on the $p{=10}^{-1.3}$ contour line, predicted by bivariate Weibull technique. 95% CI produced by Gaidai method included bivariate point, utilized by bivariate Weibull method^20–23^. High-dimensionality (say, above 2D) of biological and health systems makes it challenging to produce accurate multivariate predictions, based on available relatively limited clinical raw datasets. Hence above-described novel health system reliability approach, has advantages of optimally utilizing clinical measured datasets, while taking into account biosystem’s high dimensionality.

The Poincare type plot may be used to analyze intrinsic data structural patterns, for example 2nd order difference plot (SODP) can be used to start with. For consecutive differences, 2nd order SODP may be used to statistically observe raw timeseries data^24^.

**Fig. 4:**
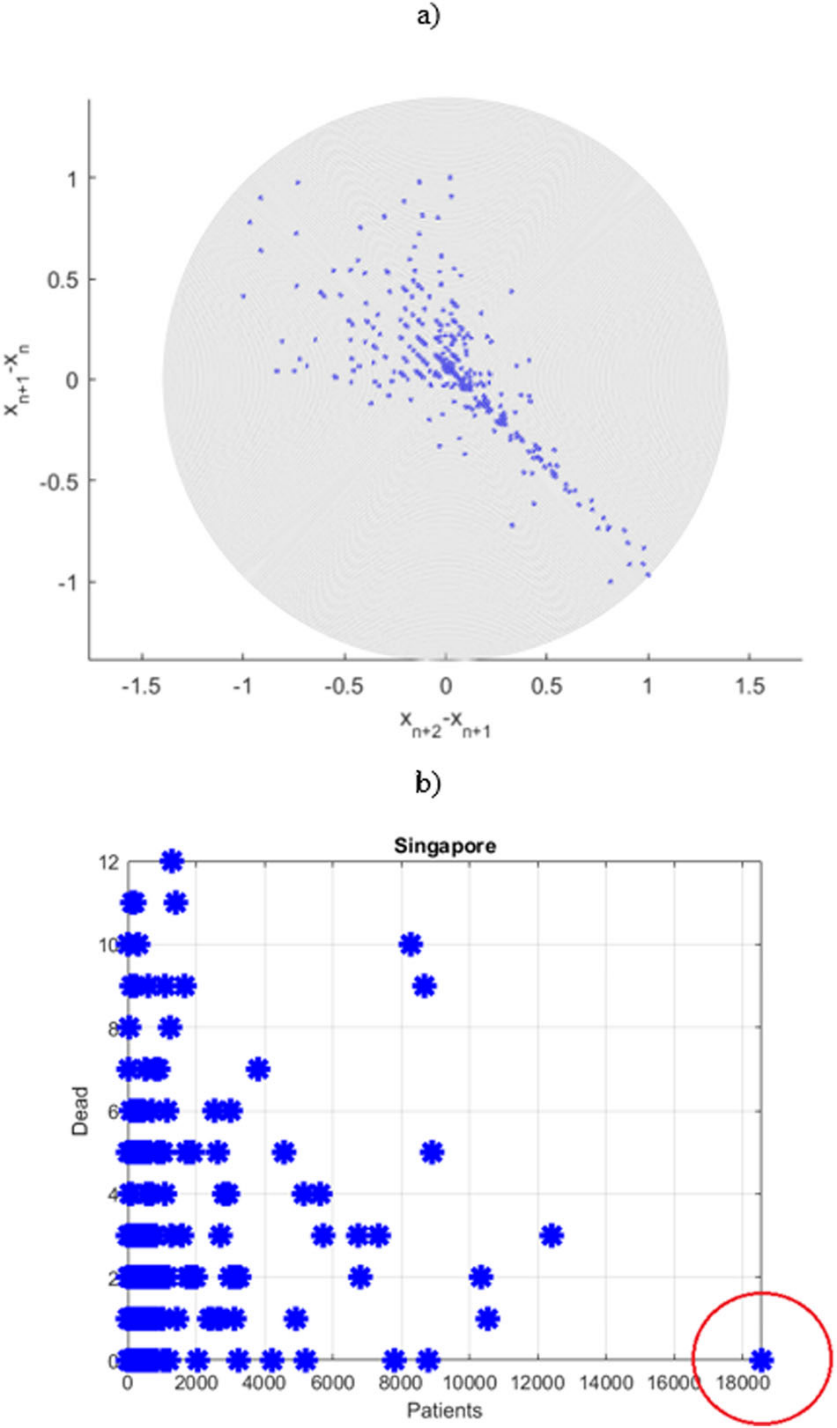
Data pattern analysis. **a** Singapore COVID-19 data 2nd order SODP plot, indicating unnatural correlation pattern. **b** Correlation between COVID-19 daily recorded new patient and deceased numbers. Circle marks outlier.

Figure 4a presents 2nd order SODP plot. When employing an entropy-based AI (artificial intelligence) recognition approach, 2nd order SODP plots may be used to spot data patterns and compare them to other similar datasets^14,15,25–27^. This study did not focus on AI pattern analysis as such, therefore Fig. 4a can be seen as motivating for further research, when underlying raw dataset quality remains an open issue.

Figure 4b demonstrates correlation between the daily number of COVID-19 fatalities and newly daily-registered patients. It is clear from Fig. 4b that raw daily recorded new patient counts contain outliers. Traditional health systems reliability techniques that deal with observed raw timeseries do not have an advantage of dealing with high-dimensional (above 2D) systems, along complex cross-correlation between different biosystem components. The key advantage of Gaidai method being its ability to assess reliability of high-dimensional non-linear dynamic biosystems.

## Discussion

Traditional timeseries reliability approaches do not always have advantage of easily handling high-dimensional dynamic systems along with cross-correlations between different key system components. Fundamental advantage of Gaidai method being its ability to examine reliability of high-dimensional dynamic bio-systems. In this investigation, synthetic wind speeds were used as validation case, as in this case analytical solution are known. The theoretical rationale of the proposed approach being thoroughly discussed. Although using direct measurement or Monte Carlo simulation to analyze the reliability of dynamic bio-systems is often appealing, it should be noted that the complexity and high-dimensionality of dynamic bio-systems require development of novel, accurate, and robust techniques that can handle available raw datasets, while utilizing them optimally.

This study’s methodology has already been shown successful when applied to a number of simulation models, but only for one-dimensional system components. Overall, quite accurate forecasts have been made. The main goal of this study was to develop a general-purpose, trustworthy, and user-friendly multi-dimensional reliability strategy. Gaidai bio-reliability method was compared to the bivariate Weibull method, using both analytically produced synthetic data and actual raw clinical data. To summarize, suggested methodology may be applied to a wide range of biological and public health studies. Presented national public health example by no means limits potential uses of advocated methodology.

## Data Availability

Data will be made available on request from corresponding author.
